# Psychometric assessment of the IBS-D Daily Symptom Diary and Symptom Event Log

**DOI:** 10.1007/s11136-016-1335-1

**Published:** 2016-06-24

**Authors:** Kathleen Rosa, Leticia Delgado-Herrera, Bernie Zeiher, Benjamin Banderas, Rob Arbuckle, Glen Spears, Stacie Hudgens

**Affiliations:** 1 Psychometrician, Leland, NC USA; 2 Astellas Pharma Global Development, Inc., 1 Astellas Way, Northbrook, IL 60062 USA; 3 Adelphi Values, Boston, MA USA; 4 Adelphi Values, Bollington, UK; 5 AbbVie, North Chicago, IL USA; 6 Clinical Outcomes Solutions, Tucson, AZ USA

**Keywords:** Patient-reported outcome, Diarrhea-predominant IBS, IBS-D, Psychometric analysis

## Abstract

**Purpose:**

Diarrhea-predominant irritable bowel syndrome (IBS-D) can considerably impact patients’ lives. Patient-reported symptoms are crucial in understanding the diagnosis and progression of IBS-D. This study psychometrically evaluates the newly developed IBS-D Daily Symptom Diary and Symptom Event Log (hereafter, “Event Log”) according to US regulatory recommendations.

**Methods:**

A US-based observational field study was conducted to understand cross-sectional psychometric properties of the IBS-D Daily Symptom Diary and Event Log. Analyses included item descriptive statistics, item-to-item correlations, reliability, and construct validity.

**Results:**

The IBS-D Daily Symptom Diary and Event Log had no items with excessive missing data. With the exception of two items (“frequency of gas” and “accidents”), moderate to high inter-item correlations were observed among all items of the IBS-D Daily Symptom Diary and Event Log (day 1 range 0.67–0.90). Item scores demonstrated reliability, with the exception of the “frequency of gas” and “accidents” items of the Diary and “incomplete evacuation” item of the Event Log. The pattern of correlations of the IBS-D Daily Symptom Diary and Event Log item scores with generic and disease-specific measures was as expected, moderate for similar constructs and low for dissimilar constructs, supporting construct validity. Known-groups methods showed statistically significant differences and monotonic trends in each of the IBS-D Daily Symptom Diary item scores among groups defined by patients’ IBS-D severity ratings (“none”/“mild,” “moderate,” or “severe”/“very severe”), supporting construct validity.

**Conclusions:**

Initial psychometric results support the reliability and validity of the items of the IBS-D Daily Symptom Diary and Event Log.

## Introduction

Diarrhea-predominant irritable bowel syndrome (IBS-D) is a common and burdensome condition, especially in individuals with moderate to severe IBS-D, who suffer significantly impaired quality of life and high healthcare costs [1–3]. As no biomarkers or clinical measures of disease activity are currently available in IBS-D, diagnosis and treatment rely on direct patient report of signs and symptoms. IBS-D diagnostic criteria rely exclusively on the evaluation of symptoms, and the recently published US Food and Drug Administration (FDA) IBS guidance [4] on the design of IBS interventional trials recommends that primary endpoints in IBS-D trials be made up of patient-reported symptom assessments [5, 6].

Historically, clinical trial primary endpoints in IBS have relied on single-item assessments that ask patients to judge whether they have experienced “adequate symptom relief” or “satisfactory relief” over the entire trial [4]. The limitations of such single-item assessments of a patient’s symptom experience in terms of covering the breadth of IBS-D symptomology have been well documented [4, 7, 8]. None of these measures meet the FDA patient-reported outcome (PRO) guidance [9] in terms of content validity, nor do they adhere to the agency’s roadmap for clinical outcomes of assessment [10, 11]. In addition, the FDA no longer considers a global measure of change to be adequate as a primary endpoint [4, 9]. Consequently, the FDA’s IBS guidance highlights the need to develop multi-item, patient-reported measures in line with the agency’s PRO guidance. While there are established measures such as the IBS-Severity Scoring System (IBS-SSS) [12] and the IBS-Quality of Life Questionnaire (IBS-QOL), these historical measures were not intended to investigate benefits of treatment in the clinical setting, nor do they meet the needs of the IBS-D population (i.e., context of use) [13]. Moreover, they do not meet the rigor as set forth by the FDA PRO guidance [9]. New measures should be developed based on qualitative research with patients and must be designed to capture the cardinal symptoms of IBS-D, including abdominal pain, bowel function, and bloating [7, 8, 14].

**Fig. 1:**
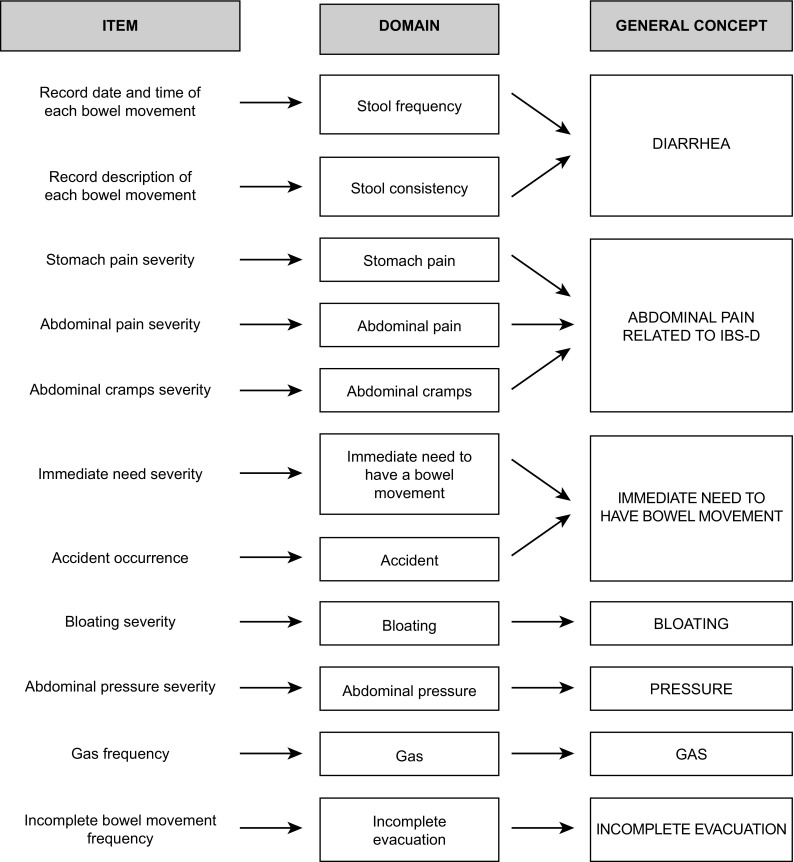
Hypothesized conceptual framework

To meet this need, the new IBS-D Daily Symptom Diary and Symptom Event Log (hereafter, “Event Log”) was developed via qualitative research among IBS-D patients, in accordance with the FDA PRO guidance [9]. A full account of the qualitative development of this instrument is provided elsewhere [15]. This prior research provides evidence that the instrument’s items demonstrate content validity and assess the full measurement continuum. The hypothesized conceptual framework based on the qualitative research is provided in Fig. 1.

Once established through qualitative research that a new PRO measures all concepts that are clinically relevant and important to patients, in a manner that patients understand and can respond to, the next step is to evaluate the initial measurement properties of the instrument. This evaluation can then be used to develop a scoring algorithm, as well as guide potential item deletion. This article presents initial results generated from a US-based, multicenter, non-interventional observational study regarding the cross-sectional psychometric properties of the IBS-D Daily Symptom Diary and Event Log.

## Methods

### Patients

Patients with clinician-verified diagnoses of mild, moderate, or severe IBS-D (per Rome III criteria) were recruited into a US-based, multicenter, and non-interventional observational study. The targeted distribution of the sample population was ~50 patients with mild, 100 with moderate, and 50 with severe disease (based on clinician reports). Patients were recruited from general practice and gastroenterology clinics between June 2012 and November 2012 and were eligible for inclusion if they met the inclusion and exclusion criteria outlined in Table 1.

**Table 1:** Inclusion and exclusion criteria

*Inclusion criteria*
The patient is 18 years of age or older
The patient is fluent in US English and capable of comprehending and signing an informed consent form for participation
The patient has a clinician-confirmed diagnosis of IBS-D
If yes, please rate the severity of the patient’s IBS-D:
□_1_ Very mild
□_2_ Mild
□_3_ Moderate
□_4_ Severe
□_5_ Very severe
The patient has experienced IBS-D symptoms for at least 6 months prior to diagnosis
The patient has experienced recurrent abdominal pain or discomfort associated with two of the three following features, at least 3 days a month, for the last 3 months
• Improvement with defecation
• Onset associated with a change in frequency of stool
• Onset associated with a change in form (appearance) of stool
The patient has experienced loose (mushy) or watery stools (type 6 or 7 on the Bristol Stool Form Scale) in at least 25 % of bowel movements and hard or lumpy stool in fewer than 25 % of bowel movements in the absence of use of anti-diarrheals or laxatives in the last 3 months
*Exclusion criteria*
The patient has an organic disease or functional gastrointestinal syndrome, other than IBS, potentially affecting the digestive tract passage or colonic function, including structure, obstruction, or ileus
The patient has a bowel movement characterized as a Bristol Stool Form Scale of 3 or less in the last 7 days prior to enrollment
The patient has benign polyps or colonic diverticulosis judged to have an influence on the digestive tract passage or colonic function
Does the patient have a history of surgical resection of stomach, small intestine, or large intestine (excluding resection of appendix or benign polyps)?
The patient has a history of ischemic colitis, history of unexplained blood passage per rectum
The patient has uncontrolled lactose intolerance, or is the patient receiving radiotherapy for abdominal disease?
The patient has a history of drug or alcohol abuse within past year or history of major psychiatric disorders or current significant depression or anxiety
The patient has cardiovascular, pulmonary, endocrine, metabolic, hematologic, neurologic, or gastrointestinal (excluding IBS) disease
The patient has a history of thyroid dysfunction

Written informed consent was obtained from all patients prior to inclusion in the study. All study forms were approved by a centralized, independent ethics committee, in accordance with the revised Declaration of Helsinki [16]. Patients were free to discontinue participation in the study at any time.

#### Demographics and clinician IBS-D severity rating

Clinicians completed a case report form during an initial screening visit, confirming that the patient had a diagnosis of IBS-D (per Rome III criteria) and met all inclusion and exclusion criteria. Clinicians also assessed the patient’s IBS-D symptom severity using a 5-point graded scale of “very mild,” “mild,” “moderate,” “severe,” or “very severe.” Once patients were screened into the study, they were asked to complete a baseline demographic and health information form.

### Patient-reported outcomes

To evaluate the newly developed symptom diary, several well-established PRO instruments were included in the study, including generic and disease-specific measures of symptoms and impact on health-related quality of life (HRQoL). Patients completed seven PRO instruments: the newly developed IBS-D Daily Symptom Diary and Event Log (described below) [15]; IBS-SSS [12]; the 12-item Short-Form Health Survey (SF-12) [17]; IBS-QOL instrument [13]; the Patient Global Impression of Severity (PGI-S); and 24-h recall and 7-day recall versions of the Patient Global Impression of change (PGI-C) [18]. The IBS-SSS, the IBS-QOL, the SF-12, the PGI-S, and the PGI-C are described in Table 2.

**Table 2:** Other instruments

PRO Name	Number of Items and item content	Scoring
IBS-SSS [12]	Five items assessing abdominal pain, abdominal distension, bowel dysfunction, and quality of life/global well-being as reported by patients.	The IBS-SSS total score is calculated by summing the five item scores, resulting in a total score ranging from 0 to 500, with higher scores reflecting higher severity of IBS The IBS-SSS has no specified recall period
SF-12 [17]	Twelve items assessing dimensions: physical functioning, role physical, role emotional, bodily pain, general health, vitality, social functioning, and mental health.	Scores for each dimension are obtained by summing the corresponding item values. The resulting scores are then rescaled from 0 (worst possible health state measured by the questionnaire) to 100 (best possible health state) In addition, two summary scores, the physical component scale (PCS-12) and the mental component scale (MCS-12), are calculated The SF-12 has a recall period of 1-week
IBS-QOL [13]	Thirty-four items assessing quality of life with impact being assessed across eight domains: dysphoria (eight items), interference with activity (seven items), body image (four items), health worry (three items), food avoidance (three items), social reaction (four items), sexual (two items), and relationships (three items)	All domain scores are converted to a 0 to 100 scale, with high scores representative of a better health state The summed global score is transformed to a 0-100 scale ranging from 0 (poor quality of life) to 100 (maximum quality of life) The IBS-QOL has a recall period of 4 weeks
PGI-S [18]	Item assessing the severity of IBS-D	Severity is measured on a scale of 1 (none) to 5 (very severe) scale The PGI-S has a recall period of “currently”
PGI-C [18]	Item assessing level of change in IBS-D	Change is measured on a scale of 1 (very much improved) to 7 (very much worse) The PGI-C has a recall period of 1 week

#### IBS-D Daily Symptom Diary

The IBS-D Daily Symptom Diary is a 7-item PRO diary measuring abdominal pain, stomach pain, abdominal pressure, bloating, abdominal cramping, frequency of gas, and the occurrence of accidents. Five of the symptoms are rated on an 11-point numerical rating scale with 0 representing absence of symptoms and 10 representing severe symptoms. Frequency of gas is measured on an ordinal scale from “none of the time” to “all of the time,” and the occurrence of accidents has a “Yes/No” response option. The recall period for all items is the past 24 h. Daily and weekly (i.e., means) scores were utilized for each item for analyses.

#### IBS-D Symptom Event Log

The IBS-D Symptom Event Log consists of three questions which ask the patient to rate, with respect to each individual bowel movement, the immediacy of need (1 = “no immediate need” to 5 = “extreme immediate need”), consistency of the bowel movement (pictorial 8-point scale: 1 = “like marbles or hard rocks” to 8 = “just liquid”), and whether the bowels were completely emptied (“Yes/No”). All items are completed after each bowel movement, and the date and time of each bowel movement were recorded. Daily and weekly (i.e., means) scores were utilized for each item for analyses.

#### Administration of PRO instruments

All data were collected on a paper case report form. Center personnel were trained to instruct patients in a standardized way to reduce data collection errors and enhance questionnaire completion compliance. Clinician severity ratings and patient demographic and health information were collected at baseline (day 1). The PRO measures administered in the study were assessed across two intervals: period 1 (study weeks 1 and 2; days 1–14) and period 2 (study weeks 3 and 4; days 15–28). The schedule of assessment is summarized in Table 3.

**Table 3:** Schedule of assessments

Protocol activities and forms to be completed	Screening day –14 ± 2	Period 1														Period 2													
		1	2	3	4	5	6	7	8	9	10	11	12	13	14	15	16	17	18	19	20	21	22	23	24	25	26	27	28
Patient Information and Consent Form (to release medical information)	✓																												
Case Report Form (clinician)	✓																												
Demographic and Health Information Form		✓																											
IBS–D Daily Symptom Diary		✓	✓	✓	✓	✓	✓	✓	✓	✓	✓	✓	✓	✓	✓	✓	✓	✓	✓	✓	✓	✓	✓	✓	✓	✓	✓	✓	✓
IBS–D Symptom Event Log (if applicable)		✓	✓	✓	✓	✓	✓	✓	✓	✓	✓	✓	✓	✓	✓	✓	✓	✓	✓	✓	✓	✓	✓	✓	✓	✓	✓	✓	✓
IBS–SSS									✓																				✓
IBS–QOL									✓																				✓
SF–12									✓																				✓
PGI–C Week																													✓
PGI–C Day																✓													
PGI–S		✓							✓							✓							✓						✓

All data were manually entered into a password-protected database; standards of quality control, including proportional double data entry, were observed. Date and time of completion were captured for the daily diary. All eligible patients who completed at least one item of the IBS-D PRO instruments at day 1, period 1 were included in the analysis population.

### Statistical methods

Psychometric analyses were performed to evaluate the item-level measurement properties of the instrument. Demographic and health information of the study population was summarized using descriptive statistics. Continuous variables were described by presenting the frequency, mean, standard deviation, median, minimum, maximum, and instances of missing data. Categorical variables were described by presenting the number and percentage of patients in each category and the number of missing data (the percentage in each category was calculated including the proportion of patients with missing values). Quality of completion was assessed for the IBS-D Daily Symptom Diary and Event Log at day 1 through day 15 as the number and percentage of patients missing responses. Items with missing data >10 % were flagged and considered candidates for deletion. If >10 % of patients endorsed the lowest or highest categories on a given item on the IBS-D Daily Symptom Diary and Event Log, the item was investigated for floor or ceiling effects, respectively. Floor or ceiling effects that are too pronounced could interfere with the ability of the score to detect improvement, deterioration, or difference between groups in a clinical trial; however, floor and ceiling effects must be interpreted in the context of the study sample and the condition being studied.

Inter-item correlations were evaluated for the IBS-D Daily Symptom Diary and Event Log items. Correlations >0.80 suggested potential redundancy and thus potential candidates for deletion [19].

The emphasis in a psychometric study is on evaluation of the magnitude of relationships between variables and the overall pattern of results rather than significance testing. As such, no adjustments are generally used for multiplicity of tests. For many psychometric analyses, significance tests are not traditionally used. Where specific significance tests are used, the threshold for statistical significance was *p* < 0.05. Statistical analyses were performed for the study using Statistical Analysis System version 9 (SAS Institute, Cary, NC, USA).

#### Psychometric evaluation of the instrument: reliability

Test–retest reliability measures the stability of a score over multiple administrations of an instrument to the same patient [20]. The time period for assessment is critical in chronic, symptomatic, or event-driven conditions because response variability may be high due to the nature of the disease. In this study, test–retest reliability was assessed by comparing 7-day average scores for the IBS-D Daily Symptom Diary and Event Log items between study weeks 1 and 4. The subgroup of stable patients for this analysis was determined using the PGI-C Week assessment at study week 4. Patients who responded “no change” on this measure were included in the test–retest analysis population. The intraclass correlation coefficient (ICC) was used to evaluate test–retest reliability, with ICCs >0.70 considered evidence of acceptable reliability [20].

#### Psychometric evaluation of the instrument: construct validity

The construct validity of the IBS-D Daily Symptom Diary and Event Log was examined via assessment of concurrent and clinical/known-groups validity.

Concurrent validity was assessed via evaluating correlations of the IBS-D Daily Symptom Diary and Event Log weekly average item scores with the IBS-SSS (no specified recall period), IBS-QOL (4-week recall period), and SF-12 (1-week recall period) at day 8. Spearman correlation coefficients were calculated and described as strong (0.60), moderate (0.40), or low (0.30) [21]. Low to moderate correlations were expected between IBS-D Daily Symptom Diary items and SF-12 scores, and moderate to high correlations were expected between IBS-D Daily Symptom Diary items and IBS-QOL and IBS-SSS scores.

Clinical (or known-groups) methods is a measure of the ability of items to discriminate between patient subgroups expected to respond differently based on severity of their condition [22]. Clinician-reported IBS-D severity at baseline was the primary classification variable used for the assessment of clinical validity [22]. Secondary measures used to define comparison groups for clinical validity assessment in the present study included the PGI-S at day 8 and the derived presence or absence of flare on day 1. Flare on a given day was defined by three or more bowel movements recorded in the IBS-D Symptom Event Log with a rating of 7 or 8 on the pictorial scale and an immediate need rating of moderate or greater. A “non-flare day” was defined as fewer than three bowel movements recorded in the IBS-D Symptom Event Log with a rating <7 on the pictorial scale and an immediate need rating of less than moderate. Analysis of variance and *t* tests were used to compare differences among groups, with differences considered significant if <0.05 level.

## Results

### Study population

A total of 202 patients (132 females; 65.3 %) were enrolled in the study, with 200 patients completing the study. Based on patient self-report, the majority of patients had moderate IBS-D (*n* = 106, 52.5 %) and the remaining patients were mostly distributed between mild (*n* = 46, 22.8 %) and severe (*n* = 44, 21.8 %) symptoms, with few reporting very mild (*n* = 5, 2.5 %) or very severe (*n* = 1, 0.5 %) symptoms. The mean age of the patient population was 46.3 years ± 14.4 (range 18–79 years), and the majority of patients were Caucasian (*n* = 122, 60.4 %) and had a high school diploma or some college or other educational certification (*n* = 126, 62.4 %). Additional demographic information is provided in Table 4.

**Table 4:** Demographic characteristics at baseline (day 1)

Characteristic	Total (clinical) sample (*N* = 202)
Age	
Mean (SD)	46.3 (14.4)
Min–Max	18.0–79.0
Gender	
Female, *n* (%)	132 (65.3)
Race	
White/Caucasian, *n* (%)	122 (60.4)
Black/African-American, *n* (%)	38 (18.8)
Native Hawaiian/Pacific Islander, *n* (%)	3 (1.5)
Other, *n* (%)	39 (19.3)
Ethnicity	
Not Hispanic/Latino, *n* (%)	176 (87.1)
Hispanic/Latino, *n* (%)	26 (12.9)
Education	
High school diploma (or GED) or less, *n* (%)	45 (22.3)
Some college or certification program, *n* (%)	81 (40.1)
College or university degree (2- or 4-year), *n* (%)	56 (27.7)
Graduate degree, *n* (%)	16 (7.9)
Other, *n* (%)	2 (1.0)
Missing/No response, *n* (%)	2 (1.0)
Patient rating of current severity of diarrhea-specific IBS	
Very mild, *n* (%)	5 (2.5)
Mild, *n* (%)	46 (22.8)
Moderate, *n* (%)	106 (52.5)
Severe, *n* (%)	44 (21.8)
Very severe, *n* (%)	1 (0.5)

### IBS-D Daily Symptom Diary and Event Log measurement properties

#### Descriptive statistics

Overall, patients were compliant in their completion of the IBS-D Daily Symptom Diary and Event Log, with only 12 patients (5.9 %) with at least one missing item on any of the 15 study days in which quality of completion was tested. Specifically, only 5.9 % of patient had any missing diary data during the 15-day period, with no more than 2 items being missed by a single patient on any particular day. No patient missed items every day, nor was there a pattern of a single item being missed. In addition, item-level missing data were very low, with no items having more than ~4.0 % missing data. Thus, quality of completion indicated there were no patient- or item-specific issues causing missing data and results did not suggest any specific item as a candidate for deletion. Patients utilized the full response scale on all items, and average symptom severity on the IBS-D Daily Symptom Diary items ranged between 3.6 and 4.1 across the items at the baseline (day 1) assessment, with 26.3 % of patients reporting gas most or all of the time and only 7.4 % experiencing an accident on day 1. Patients reported an average of three events (bowel movements) at baseline (day 1) on the IBS-D Symptom Event Log, with complete emptying ~54 % of the time, a mean stool consistency of 5 (“soft chunks or clumps”), and a mean immediacy rating of 3 (“moderate immediate need”). One patient, recruited with severe disease, reported 42 episodes in a day, which was confirmed upon qualitative review of the source data.

Review of the floor and ceiling effects revealed that 5–17 % of the patient population chose the lowest possible response for a particular item on day 1. Floor effects greater than the 10 % a priori criterion were present for all of the daily symptom diary items except frequency of gas; however, given that the highest percentage scoring at floor for any one item was 17 %, the floor effects were considered marginal. No ceiling effects (>10 % scoring at ceiling) were observed for any of the Daily Symptom Diary items.

#### Inter-item correlations within the IBS-D Diary

Inter-item correlations were examined using data from day 1 (Table 5). With the exception of correlations with the frequency of gas and accidents items, moderate to high inter-item correlations were observed among all items of the IBS-D Daily Symptom Diary and Event Log (day 1 range 0.67–0.90; Table 5). The inter-item correlations were highest between the two items measuring severity of abdominal pain and stomach pain (*r* = 0.90 for the daily report at day 1). These two items appear to be redundant, suggesting one can be deleted. The correlations of these two items with the items asking about abdominal cramps and abdominal pressure were also close to or above 0.80, suggesting all of these abdominal symptoms are closely related. Of note, point bi-serial correlation coefficients were generated between the IBS-D Daily Symptom Diary item 7 (accidents) and IBS-D Daily Symptom Diary at day 8. The range of correlations was low (range 0.19–0.28). This finding is likely due to the very low frequency of report for accidents on a given day. As larger clinical datasets become available, it will be of value to further investigate the relationship between this item and others by using a known-groups approach and by evaluating the variable over a longer period of time than 1 day.

**Table 5:** Inter-item correlations–Spearman correlations of IBS-D daily symptom diary items at day 1

IBS-D daily symptom diary item	IBS-D daily symptom diary item					
	Abdominal pain	Stomach pain	Abdominal cramps	Abdominal pressure	Bloated	Frequency of gas
Abdominal pain	1.000	–	–	–	–	–
Stomach pain	0.904	1.000	–	–	–	–
Abdominal cramps	0.824	0.807	1.000	–	–	–
Abdominal pressure	0.862	0.822	0.789	1.000	–	–
Bloated	0.748	0.724	0.674	0.790	1.000	–
Frequency of gas	0.362	0.370	0.310	0.382	0.462	1.000

#### Test–retest reliability

Test–retest reliability was evaluated by comparing 7-day average scores of individual items on the IBS-D Daily Symptom Diary and Event Log between weeks 1 and 4, among 115 stable patients who reported “no change” from baseline in their symptoms on the PGI-C Week at study week 4. All abdominal symptom items except frequency of gas met the threshold for test–retest reliability (ICC ≥ 0.70), with ICC scores from 0.78 to 0.83. The ICC for frequency of gas was 0.66, marginally below the threshold. Item 7 (accidents) used a Yes/No dichotomous scale, and therefore, Cohen’s kappa statistics were generated for a single-day score rather than weekly scores. Reliability results for item 7 were well below the threshold; however, these results are reported only between day 22 and day 28. Accidents on a given day are reported with very low frequency and daily symptoms are highly variable, both of which will weaken the ICC.

For the IBS-D Symptom Event Log, the mean number of events also met or surpassed the threshold of 0.70; however, mean immediacy (ICC = 0.64) and stool consistency (ICC = 0.66) narrowly missed the 0.70 threshold. The ICC score for the incomplete evacuation question fell short of the threshold (ICC = 0.46) (Table 6).

**Table 6:** Test–retest reliability—study week 1 and study week 4 (stable group)

PRO score	*N*	Reliability–ICC (95 % confidence interval)
IBS-D Daily Symptom Diary weekly mean scores		
Mean abdominal pain	115	0.778 (0.695–0.841)
Mean stomach pain	115	0.789 (0.708–0.849)
Mean abdominal cramps	115	0.795 (0.717–0.854)
Mean abdominal pressure	115	0.813 (0.740–0.866)
Mean bloated	115	0.834 (0.769–0.882)
Mean frequency of gas	115	0.655 (0.537–0.748)
Accidents^a^	160	0.174 (–0.165 to 0.513)
IBS-D Symptom Event Log weekly mean scores		
Mean total events	110	0.834 (0.766–0.883)
Mean immediacy	110	0.642 (0.518–0.740)
Mean consistency	110	0.659 (0.539–0.753)
Mean percent of completely empty bowels	110	0.455 (0.294–0.591)

^a^ Test-retest was run for the “accidents” item between days 22 and 28

#### Construct validity: correlations between symptoms and events (concurrent validity)

Moderate correlations were observed between immediacy of need and all abdominal items (range 0.50–0.56) except frequency of gas (0.32) (Table 7). The number of daily events had small to moderate correlations with abdominal pain (0.40) and abdominal cramps (0.41) but smaller correlations with stomach pain (0.34) and bloating (0.29). Mean stool consistency had small to moderate correlations with all abdominal items except frequency of gas, where the correlation was negligible (0.13). However, all abdominal symptoms demonstrated very low correlations with daily percentage of completely emptied bowels. Frequency of gas yielded a low correlation with all event log items (Table 7). Accidents at day 8 (results not shown) also yielded low correlations with the event log items (range −0.08 to −0.27).

**Table 7:** Spearman correlations between the IBS-D daily symptom diary and event log at week 1

Event log	IBS-D Daily Symptom Diary item^a^					
	Abdominal pain	Stomach pain	Abdominal cramps	Abdominal pressure	Bloated	Frequency of gas
Mean number of daily events	0.397	0.336	*0.408*	0.375	0.292	0.219
Average daily mean immediacy of need	*0.526*	*0.499*	*0.521*	*0.558*	*0.545*	0.321
Average daily mean consistency of the bowel movement	0.398	0.361	*0.401*	*0.411*	0.354	0.128
Mean daily percentage of completely emptied bowels	−0.161	−0.136	−0.144	−0.127	−0.231	−0.242

^a^Italics indicates moderate correlations

#### Construct validity: correlations with generic and disease-specific measures (concurrent validity)

A logical pattern of correlations was also observed between IBS-D Daily Symptom Diary items and the concurrent scores at day 8 (Table 8). As hypothesized, correlations among the domains of the SF-12 and the IBS-D Daily Symptom Diary items were extremely low for all SF-12 domains except “bodily pain,” which was moderately correlated with the IBS-D Daily Symptom Diary items related to abdominal pain, stomach pain, and abdominal cramps (range −0.42 to −0.48). Correlations were not calculated for the accidents item.

**Table 8:** Spearman correlations of IBS-D daily symptom diary at day 8 (a) IBS-QOL and (b) SF-12

Concurrent measures	IBS-D Daily Symptom Diary item^a^					
	Item 1	Item 2	Item 3	Item 4	Item 5	Item 6
	Abdominal pain	Stomach pain	Abdominal cramps	Abdominal pressure	Bloated	Frequency of gas
IBS-QOL^b^						
Dysphoria	−0.452	−0.465	−0.458	−0.463	−0.497	−0.303
Interference with activity	−0.427	−0.455	−0.442	−0.437	−0.478	−0.283
Body image	−0.424	−0.403	−0.411	−0.436	−0.55	−0.334
Health worry	−0.350	−0.414	−0.356	−0.406	−0.426	−0.248
Food avoidance	−0.364	−0.405	−0.370	−0.403	−0.428	−0.257
Social reaction	−0.410	−0.419	−0.379	−0.397	−0.464	−0.320
Sexual	−0.308	−0.330	−0.319	−0.309	−0.323	−0.267
Relationships	−0.413	−0.392	−0.417	−0.391	−0.391	−0.303
Overall	−0.469	−0.486	−0.470	−0.481	−0.532	−0.347
Total score	0.553	0.552	0.525	0.542	0.573	0.319
SF-12^c^						
Physical functioning	−0.111	−0.136	−0.088	−0.112	−0.159	−0.166
Role physical	−0.269	−0.286	−0.254	−0.271	−0.277	−0.262
Bodily pain	−0.423	−0.475	−0.423	−0.377	−0.372	−0.302
General health	−0.155	−0.154	−0.114	−0.136	−0.102	−0.147
Vitality	−0.159	−0.128	−0.159	−0.204	−0.198	−0.179
Social functioning	−0.311	−0.298	−0.269	−0.286	−0.318	−0.353
Role emotional	−0.210	−0.216	−0.232	−0.285	−0.297	−0.266
Mental health	−0.245	−0.208	−0.260	−0.284	−0.302	−0.247
Physical component scale	−0.240	−0.289	−0.201	−0.187	−0.198	−0.204
Mental component scale	−0.231	−0.199	−0.239	−0.283	−0.313	−0.272

Spearman correlation coefficients were generated between the IBS-D Daily Symptom Diary and concurrent measures

^a^IBS-D Daily Symptom Diary Items 1–5 are scored on a numeric rating scale from 0 to 10, with higher scores representing more severe symptoms. IBS-D Daily Symptom Diary Item 6 is scored on a five choice ordinal scale with higher scores representing more frequent gas

^b^IBS-QOL scores range from 0 to 100, with higher scores representing better health states. The IBS-SSS total score ranges from 0 to 500 with higher scores reflecting higher severity of IBS

^c^SF-12 scores range from 0 to 100, with higher scores representing better health states

As expected for the disease-specific measures, moderate correlations were observed between IBS-QOL domains and the individual symptom items of the IBS-D Daily Symptom Diary, except the IBS-QOL sexual domain, for which correlations were low (range −0.27 to −0.33). Also as expected, the IBS-D Daily Symptom Diary items all correlated most highly with the only concurrent symptom measure, the IBS-SSS, ranging from 0.53 to 0.57 for all items except frequency of gas, which was correlated with the IBS-SSS at 0.32 but which was found to have low correlations with all concurrent domains. Overall, a logical pattern of correlations supported the validity of the Daily Symptom Diary items as measures of IBS symptoms.

#### Construct validity: clinical (known-groups) validity

**Fig. 2:**
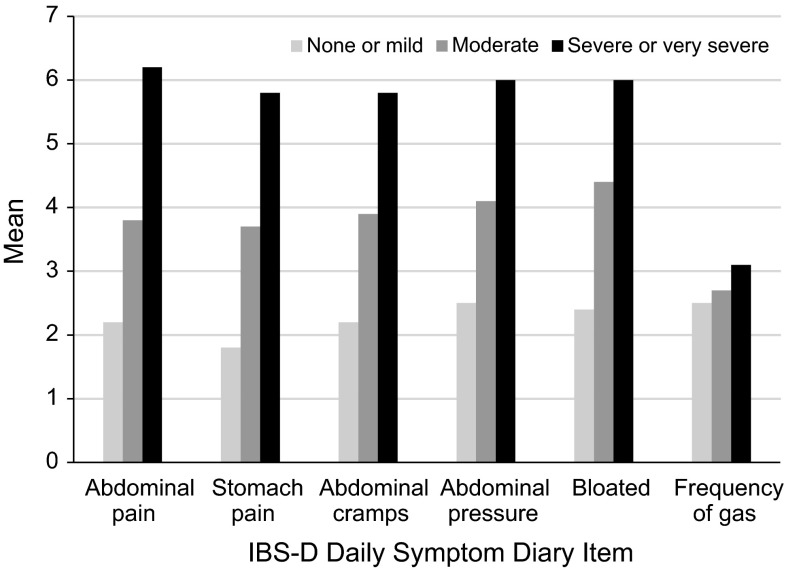
IBS-D Daily Symptom Diary by PGI-S response at day 8. IBS-D Daily Symptom Diary Items 1–5 are scored on a numeric rating scale from 0 to 10, with higher scores representing more severe symptoms

Statistically significant differences in each of the IBS-D Daily Symptom Diary item scores were observed between groups defined by patients’ ratings on the PGI-S as none/mild, moderate, or severe/very severe. All IBS-D Daily Symptom Diary item scores increased monotonically across the PGI-S-defined groups, indicating that patients reporting worse global severity ratings also had worse symptoms scores on the diary (Fig. 2). With regard to the accidents item (results not shown), a greater number and percentage of patients in the severe/very severe group (*n* = 26, 60.47 %) reported having accidents compared with the moderate group (*n* = 20, 21.05 %), with the fewest number of patients reporting accidents in the none/mild group (*n* = 6, 10.71 %). Despite the difference in the day of data collection (day 8 for the PGI-S vs. the 7 days prior for the accident item), these results are quite strong, indicating item 7 should be evaluated carefully in future analyses with an eye toward how it might best be incorporated into scoring with the ordinal rating scale items.

**Fig. 3:**
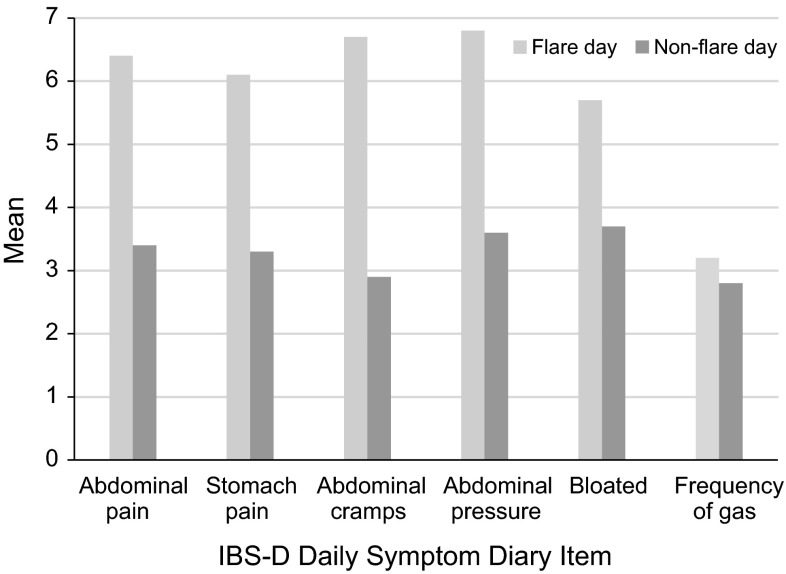
IBS-D Daily Symptom Diary by flare experience at day 1

Patients experiencing a flare day reported significantly higher symptom severity on each of the IBS-D Daily Symptom Diary items except for frequency of gas (Fig. 3).

## Discussion

The goal of this study was to psychometrically assess the initial, item-level measurement properties of the IBS-D Daily Symptom Diary and Event Log items.

The descriptive statistics showed minimal missing data and good response distributions, suggesting the response scales were fully utilized. Floor effects were slightly higher than predicted for all items except one, but still considered acceptable given that they were evaluated using data from a single day; IBS-D symptoms are highly variable and high symptom severity is not expected as a daily occurrence. The proportion of subjects scoring at ceiling was minimal.

Test–retest reliability of the IBS-D Daily Symptom Diary and Event Log met threshold for all items with the exception of gas (ICC = 0.66), mean immediacy (ICC = 0.64), stool consistency (ICC = 0.66), and incomplete evacuation (ICC = 0.46) items. As the ICC scores for gas, immediacy, and stool consistency only narrowly failed to meet the 0.70 threshold, these results are not considered of concern because these are highly variable symptoms. The low ICCs for incomplete evacuation and accidents are of greater concern and should be reanalyzed as additional data become available. In particular, given the very low frequency of report of accidents on a given day, test–retest reliability for accidents should be evaluated in a larger clinical sample by looking at periods of time that will be used as endpoints rather than looking at single-day occurrences.

Inter-item correlations were highest between the two items measuring severity of abdominal pain and stomach pain, suggesting that the items are measuring very similar concepts and are possibly redundant. This finding provides evidence that patients may think that abdominal pain and stomach pain are the same concept, which is consistent with qualitative data in which patients were thinking about the same part of their body when responding to these items [15]. With regard to the qualitative data, six of the 11 subjects in the cognitive debriefing interviews stated that the stomach pain and abdominal pain items were the same, while only three reported a difference [15]. Further, when patients indicated location on a diagram, there was no consistent indication that patients made a distinction between abdomen and stomach. Finally, given that cramping and pressure items use the term “abdominal” and considering that both pain items were well understood during qualitative testing (i.e., cognitive debriefing), the “abdominal pain” item will be retained and the “stomach pain” item removed [15].

The abdominal pain and stomach pain items were also relatively closely related to the abdominal cramps and abdominal pressure items. It could be argued that retention of just the abdominal pain item is sufficient and that the other three do not add great additional value. However, the abdominal cramps item seemed to discriminate best between flare and non-flare days in the known-groups analyses, and so arguably it provides valuable additional information. Therefore, all three items (abdominal pain, cramps, and pressure) will be retained for further testing.

Evaluation of concurrent validity demonstrated a logical pattern of correlations with concurrent measures, which supports the validity of the IBS-D Daily Symptom Diary and Event Log items as measures of IBS-D symptoms. In accordance with predictions, the IBS-D Daily Symptom Diary showed mostly low correlations with the HRQoL questionnaires and higher, moderate correlations with the IBS-SSS. Furthermore, the instrument correlated more highly with the disease-specific IBS-QOL than with the generic SF-12. The lack of overlap in recall periods between the IBS-D Daily Symptom Diary and Event Log and the concurrent measures is likely to have been a factor in reducing the magnitude of some correlations. For example, the IBS-QOL has a 4-week recall period, and thus, it is perhaps not surprising that the correlation with a week average of IBS-D Daily Symptom Diary item scores was low in addition to content differences (i.e., a symptom-based measure vs. a quality of life-based measure). Similar content between the IBS-D Symptom Diary and the IBS-SSS is the reasoning behind moderate correlations. Overall, these findings support the concurrent validity of the instrument.

For all items on the IBS-D Daily Symptom Diary and Event log, statistically significant differences in each of the IBS-D Daily Symptom Diary item scores were observed between groups defined by patients’ ratings on the PGI-S, indicating that those patients with more severe IBS-D are responding on the more severe end of the questionnaire’s spectrum. These results provide strong evidence that the different items of the IBS-D Daily Symptom Diary and Event Log are able to discriminate among patients of differing severity.

The findings reported here, as well as the previously conducted qualitative research, provide strong evidence in support of the initial psychometric validity of the IBS-D Daily Symptom Diary 24-h recall questions and the IBS-D Symptom Event Log questions.

The next step is the creation of summary scores assessing abdominal and bowel symptoms; this work is ongoing.
